# Imaging and Clinical Factors Associated With Return to Play and Recurrence After Quadriceps Muscle Strain Injury

**DOI:** 10.1177/19417381261476889

**Published:** 2026-08-27

**Authors:** Samuel Pietsch, Brady Green, Anthony G. Schache, Andrew Rotstein, Milo MacBain, Michael Paks, Tania Pizzari

**Affiliations:** † La Trobe Sport and Exercise Medicine Research Centre, School of Allied Health, Human Services and Sport, College of Science, Health and Engineering, La Trobe University, Melbourne, Victoria, Australia; ‡ School of Health Sciences and Physiotherapy, University of Notre Dame Australia, Fremantle, Western Australia, Australia; § School of Allied Health, Human Services and Sport, La Trobe University, Melbourne, Victoria, Australia; ‖ Victoria House Medical Imaging, I-Med Radiology Network, Melbourne, Victoria, Australia; ¶ Monash Health/Monash University, Melbourne, Victoria, Australia

**Keywords:** MRI, muscle strain, quadriceps injury, rectus femoris, reinjury, return to play

## Abstract

**Background:**

Limited information is available about factors that impact prognosis after quadriceps muscle strain injury, particularly injuries involving the rectus femoris. This study aims to determine whether baseline clinical and radiological findings are associated with time to return to play (RTP) and injury recurrence after rectus femoris muscle strain injury in elite Australian Football players.

**Hypothesis:**

Baseline magnetic resonance imaging (MRI) findings related to the specific anatomical location and severity of rectus femoris injury will influence time to RTP and recurrence.

**Study Design:**

Cross sectional study.

**Level of Evidence:**

Level 4.

**Methods:**

A total of 163 quadriceps muscle strain injuries from 127 elite Australian Football players were analyzed. The impact of clinical (player characteristics, injury mechanism, injury type) and MRI findings (anatomical location of injury, injury severity) on RTP and injury recurrence were explored using time to event (survival) analyses.

**Results:**

RTP time frames (means and standard deviations) were longest for injuries affecting the rectus femoris proximal free tendon (50.4 ± 16.2 days) and central aponeurosis (33.7 ± 20.4 days). Central aponeurosis injuries with disruption took longer to RTP compared with central aponeurosis injuries without disruption (hazard ratio [HR] = 0.47; *P* = 0.03), and longer than injuries involving the anterior (HR = 0.59; *P* = 0.05) or posterior aponeuroses (HR = 0.56; *P* = 0.02). Increased severity of aponeurotic disruption was also associated with prolonged RTP (*P* < 0.01). Reinjuries were associated with longer RTP than index injuries (adjusted HR = 0.49; *P* = 0.04). No clinical or MRI factors were associated with injury recurrence.

**Conclusion:**

Rectus femoris injuries with significant connective tissue disruption, particularly those affecting the proximal free tendon or central aponeurosis, as well as reinjuries, may require extended time for rehabilitation and RTP.

**Clinical Relevance:**

An understanding of baseline MRI findings related to the specific anatomical location and severity of connective tissue disruption can enhance the ability of clinicians to estimate expected RTP time frames after rectus femoris injury.

Quadriceps muscle strain injuries are prevalent in elite running and kicking sports,^10,12,28,33^ and can result in extended absences from competition. These injuries can pose a significant performance and financial burden for athletes and their teams.^15,20^

Average return to training time and recurrence frequencies have been described in elite football (19.5 days, recurrence frequency 15%) and track and field (20.4 days, recurrence frequency 13.2%).^13,30^ In elite Australian Football League (AFL) players, quadriceps strains typically involved the rectus femoris and took on average 28.8 days to return to play (RTP) (range, 5 to 82 days).^39^

Clinical and radiological (e.g., magnetic resonance imaging [MRI]) findings can impact recovery outcomes after quadriceps muscle strain. Vasti injuries have shorter RTP compared with rectus femoris injuries,^7,39^ while injuries involving the rectus femoris central aponeurosis require longer rehabilitation time compared with more peripheral injuries,^1,7,30^ Quadriceps strain injuries that occur during kicking can be associated with longer recovery time frames,^39^ with injuries occurring in the proximal free tendons of the rectus femoris linked predominantly to kicking or high-velocity sprinting mechanisms.^16,23,30^

The prognostic value of acute MRI findings for predicting recovery and reinjury after muscle strain injury is debated.^22,46,51^ Studies on hamstring and calf injuries suggest injury to connective tissue structures (intramuscular tendons or aponeuroses) may be associated with prolonged recovery.^2,11,18,35,42^ Similarly, severe central aponeurosis injuries in the rectus femoris may require extended time to RTP,^1,7^ and be more prone to reinjury.^4,30^ Although recovery after rectus femoris muscle strain injuries appears to be related to the location and extent of connective tissue disruption,^14,24,31,38^ evidence exploring the association between clinical and imaging findings to prognosis after rectus femoris injury is limited.

The aims of this study were to (1) describe MRI findings and recovery time frames for quadriceps muscle strain injury according to anatomical location and severity, focusing on rectus femoris injuries; and (2) determine whether baseline clinical and MRI findings are associated with time to RTP and injury recurrence.

## Methods

### Study Procedure, Cohort and Data Collection

Ethical approval for this study was obtained from La Trobe University Human Ethics Committee (FHEC13/057). Project approval was obtained from the AFL Research Board and La Trobe University Human Ethics Committees (FHEC13/057). Written informed consent was received from all included subjects and the rights of participants were protected.

Clinical and MRI data of quadriceps muscle strain injuries were extracted from the Soft Tissue injury Registry of the AFL (STRAFL) for injuries that occurred over 9 consecutive seasons (2014-2022). Data were submitted from all 18 AFL teams (range, 13 to 18 teams participating per year). Injuries were reported to the STRAFL when a club doctor and/or physical therapist diagnosed a player clinically with a time loss (≥1 training session and/or match missed) quadriceps muscle strain injury. Injuries caused by blunt trauma (i.e., contusions) were not reported. A standardized injury report was completed by club physical therapists. The clinical data recorded in the STRAFL has been described previously,^39^ and included the following categories:

1. Player demographics: chronological age (years), height (cm), weight (kg), ethnicity (indigenous, nonindigenous), leg dominance (preferred kicking leg), lower limb injury history (previous quadriceps/hamstring muscle strain injury, hip/knee joint injury)
2. Training and match history: stage of the season, whether the player completed >80% of the prescribed preseason training program.
3. Key injury characteristics: date of injury, activity and mechanism of injury (acceleration, deceleration, high intensity running, steady state running, sudden change of direction, kicking (kicking leg), kicking (stance leg), landing, jumping, gradual onset, nil specific, other).
4. Recovery outcomes after injury: number of days to reach pain-free walking, return to running at >90% of maximum speed, return to full training, and RTP.

The number of days between the date of injury to the date of return to an official competitive match determined the RTP period. Time to reinjury was quantified as the days passed from the date of the index injury to when the subsequent injury occurred. Data for recovery outcomes were incomplete for some cases for reasons including: the end of the season occurred; the player sustained a reinjury or a different injury during rehabilitation; the player was rehabilitating a separate injury concurrently; no matches were scheduled at the time of RTP (e.g., preseason injuries); or no data were provided. Players who sustained a preseason injury and returned to full training when no competitive matches were scheduled were deemed to have achieved RTP (n = 26). If a subsequent injury occurred within the rehabilitation period, only data related to recovery milestones achieved for that case before the point of reinjury were analyzed.

All cases with MRI evidence of a quadriceps muscle strain injury (n = 163) were included for analysis.

### Data Synthesis

Each case was categorized initially as either an index injury or a reinjury. Reinjury cases were defined as those with evidence of a previous quadriceps injury occurring on the same side and involving the same primary muscle in the 2 years before the date of injury.^36^ Quadriceps muscle strain injuries that occurred before RTP (e.g., during rehabilitation) were included as reinjury cases. Reinjuries were identified through analysis of recorded data for date of injury, side of injury, and the players’ injury history. MRI reports were also reviewed to confirm date and injury location. Each injury was also categorized as either a case that went onto recur (i.e., sustain a further quadriceps injury on the same side in the same primary muscle within 2 years) or a case where no further injury occurred within the data collection period.

### MRI Classification

Two blinded radiologists reviewed 172 deidentified MRI scans independently. MRI scans were performed on either 1.5 or 3.0 Tesla scanners. Although specific parameters varied between institutions, standard soft tissue MRI protocols typically included multiplanar T1, T2, or proton density (PD)-weighted sequences combined with fluid sensitive T2 or PD-weighted fat suppressed sequences. Initial classification results were compared, and, where disagreement occurred (n = 79), consensus was achieved via discussion between radiologists. Where consensus could not be reached (n = 9), an additional senior, experienced, musculoskeletal radiologist acted as a third reviewer to resolve classification decisions and verify the overall imaging results. A total of 9 cases were excluded after MRI analysis (contusions, n = 5; incorrect muscle, n = 2; poor image quality, n = 2). Data were collected and managed using the REDCap electronic data capture tool.

MRI findings were recorded using a standardized classification system (Supplementary file 1, available in the online version of this article), which included the following:

- Muscle(s) injured (primary and/or secondary muscles injured)
- General position of primary injury within the muscle (superior/inferior, medial/lateral, superficial/deep)
- Anatomical location of injury:
  - rectus femoris (proximal free tendon: proximal direct tendon, proximal indirect tendon, proximal conjoint tendon; myoaponeurotic: anterior aponeurosis, central aponeurosis (often described as the “central intramuscular tendon”), posterior aponeurosis; myofascial/intramuscular; isolated degloving; distal quadriceps tendon)
  - vasti (musculotendinous junction, myofascial)

- Presence (yes/no) and severity of aponeurosis disruption (AD): none (0%), mild (<50%), severe (50% to 99%), complete (100%)
- Aponeurosis waviness: yes/no
- Aponeurosis retraction: yes/no and length of defect (millimeters)
- Evidence of degloving: yes/no
- Scar tissue (from a previous quadriceps muscle strain injury): yes/no and the location relative to the current injury (at site of acute injury or remote from acute injury)
- Edema length and width: craniocaudal (millimeters) and transverse width (millimeters)
- Hematoma present: intramuscular (yes/no), or intermuscular (yes/no)

If there was >1 site of injury, the anatomical location with the largest area of injury and/or highest severity of AD was classified as the primary anatomical location of injury. Myoaponeurotic injuries with no AD were considered musculotendinous injuries, whereas those with AD were comparable with tendinous injuries.^42^

### Statistical Analysis

Descriptive statistics, including frequency distributions, were generated for player demographics. These variables included past injury history (quadricep muscle strain, hamstring strain, hip or knee injury), activity, mechanism of injury, and leg involvement (dominant versus nondominant kicking leg). Data were also explored to describe and identify the cumulative incidence of reinjury. Frequency distributions were used to describe the MRI findings for the gross location of injury, primary anatomical location of injury, the presence and severity of AD, the presence of tendon/aponeurosis waviness or retraction, evidence of degloving, and the presence of intramuscular or intermuscular hematoma. Edema length and width were used to calculate the area of edema for each injury. Continuous variables were assessed for normality (Shapiro Wilk test) and outliers removed (data 3 × interquartile range [IQR] below Q1/above Q3, or there was clinical inconsistency with results), with most of the data being distributed non-normally.

Chi-square tests were used to evaluate whether associations existed between: (1) mechanism of injury versus presence of AD on MRI; and (2) mechanism of injury for index injuries versus reinjuries. Potential associations between clinical and MRI findings and time to RTP were visualized and explored. Univariate survival analyses (Kaplan-Meier survival curves, log-rank tests) including hazard ratios (HR) with 95% CIs were generated to test associations and inform multivariable models.

Variables that did not violate assumptions and showed a univariate association, along with key clinical variables identified in the literature from previous studies of muscle strain injuries (e.g., age, injury history),^17,19,40^ were included in multivariable Cox regressions to evaluate potential independent associations (adjusted HR [AHR]) with RTP. These analyses were performed for cases where the rectus femoris was the primary injury. Collinearity was avoided with exploration and analyses of related variables. Severity of AD best represented the degree of connective tissue disruption and was therefore used in models (proxy for presence of AD, waviness, retraction). Cases that went on to recur (early, within 2 months; all cases, within 2 years) were analyzed to determine whether clinical and imaging findings were associated with recurrence. A separate analysis for vasti injuries was not performed due to the sample size. Injured players who did not return to train or RTP before the end of the season (e.g., due to a secondary injury occurring) were censored to account for time loss associated with all cases. Censoring concluded at the end of the season during which the injury occurred to ensure inflated RTP periods were not recorded.^5,6^ Statistical software (IBM SPSS Statistics 30.0, Version 0) was used to perform data analyses and a significance level was set at *P* < 0.05.

## Results

### Overview and General Injury Characteristics

A total of 163 quadriceps muscle strain injuries affecting 127 players were included, with 136 cases (83.4%) categorized as index injuries and 27 cases (16.6%) categorized as reinjuries. Most cases affected the dominant kicking leg (n = 114, 69.9%), with 72 injuries (44.2%) occurring from a kicking mechanism. Over one-third (n = 53, 39.0%) of the index injury cases had a history of a previous quadriceps muscle strain injury, with 32 (24.2%) of these cases involving the same leg.

Rectus femoris was the primary muscle injured in 147 cases (90.2%). Most rectus femoris injuries were located centrally (69.4%) in the proximal (42.2%) and middle (42.9%) thirds of the muscle, with approximately half affecting both the superficial and deep parts of the muscle (53.1%) (Table 1). Only 16 (9.8%) cases were located primarily in the vasti (vastus intermedialis, n = 12; vastus medialis, n = 3; vastus lateralis, n = 1). Most vasti injuries (81.3%) were located in the middle (n = 8, 50%) or distal (n = 5, 31.3%) thirds of the thigh and usually occurred from injury mechanisms other than kicking (80% of vasti cases). Cases categorized as reinjuries affected the rectus femoris (n = 26, 96.3%) except for 1 case that affected the vastus intermedialis. (Supplementary File 2 and Table S1, available online)

**Table 1.** Primary position and anatomical location of 163 quadriceps muscle strain injuries

		Superior-inferior, N			Medial-lateral, N			Anterior-posterior, N			Edema length, mm*^a^*	AD present	Retraction	Retraction length, mm
	N	Proximal	Mid	Distal	Medial	Central	Lateral	Superficial	Deep	S&D	Mean ± SD	N	N	Mean ± SD
Rectus femoris	147	62	63	22	6	102	39	27	42	78	102.6 ± 54.1	78	20	18.9 ± 9.9
Free tendon	9	8	0	1	0	9	0	3	0	6	65.7 ± 47.1	9	6	23.7 ± 7.5
Myoaponeurotic	111	35	61	15	4	83	24	24	25	62	106.7 ± 55.8	69	10	19.9 ± 10.8
Anterior aponeurosis	24	23	1	0	2	12	10	21	0	3	91.3 ± 64.9	19	2	18.8 ± 20.9
Central aponeurosis	55	12	38	5	0	55	0	3	0	52	118.0 ± 54.5	39	8	20.2 ± 9.4
Posterior aponeurosis	32	0	22	10	2	16	14	0	25	7	99.2 ± 47.9	11	0	NA
Myofascial	21	19	1	1	2	4	15	0	17	4	87.3 ± 40.7	0	0	NA
Isolated degloving	6	0	1	5	0	6	0	0	0	6	136.4 ± 46.8	0	4	9.4 ± 1.7
Vastii	16	3	8	5	7	7	2	12	1	3	75.9 ± 48.4	0	0	NA

AD, aponeurosis disruption; S&D, both superficial and deep.

a Craniocaudal length of edema.

### Characteristics of Rectus Femoris Injury

#### Anatomical Location of Injury

Three-quarters (n = 111, 75.6%) of rectus femoris injuries were classified as myoaponeurotic. These were located at or within the central (n = 55), posterior (n = 32), or anterior (n = 24) aponeuroses. Only 9 injuries (6.1%) involved the rectus femoris free tendons (8 proximal, 1 distal). In 30 rectus femoris injuries (20.4%), a secondary muscle injury was found in the vasti (vastus lateralis [n = 15], vastus intermedialis [n = 10] and the vastus medialis [n = 5]) (Supplementary File 2 and Table S1, available online).

#### Injury Severity

Over half (n = 78, 53.1%) of rectus femoris injuries showed evidence of AD. Mild AD (<50% disruption) was found in 47 cases (32%), severe AD (50% to 99% disruption) in 22 cases (15%), and complete (100%) tendon disruption/AD was found in 9 cases (6.1%). Five cases with complete disruption involved the free tendons and 4 cases involved the central aponeurosis (Supplementary File 2 and Table S2, available online).

AD was present in 70.1% (n = 39 of 55) of injuries affecting the central aponeurosis, 79.2% (n = 19 of 24) of those affecting the anterior aponeurosis, and 34.3% (n = 11 of 32) of those affecting the posterior aponeurosis (Table 1). Waviness was observed only in cases with AD. Most (n = 26 of 31, 83.9%) cases with severe or complete AD showed waviness (Supplementary File 2 and Figure S1, available online). An intramuscular hematoma was present in 40.8% (n = 60) of cases, while an intermuscular hematoma was present in 47.6% (n = 70) of cases.

#### Mechanism of Injury

**Figure 1.**
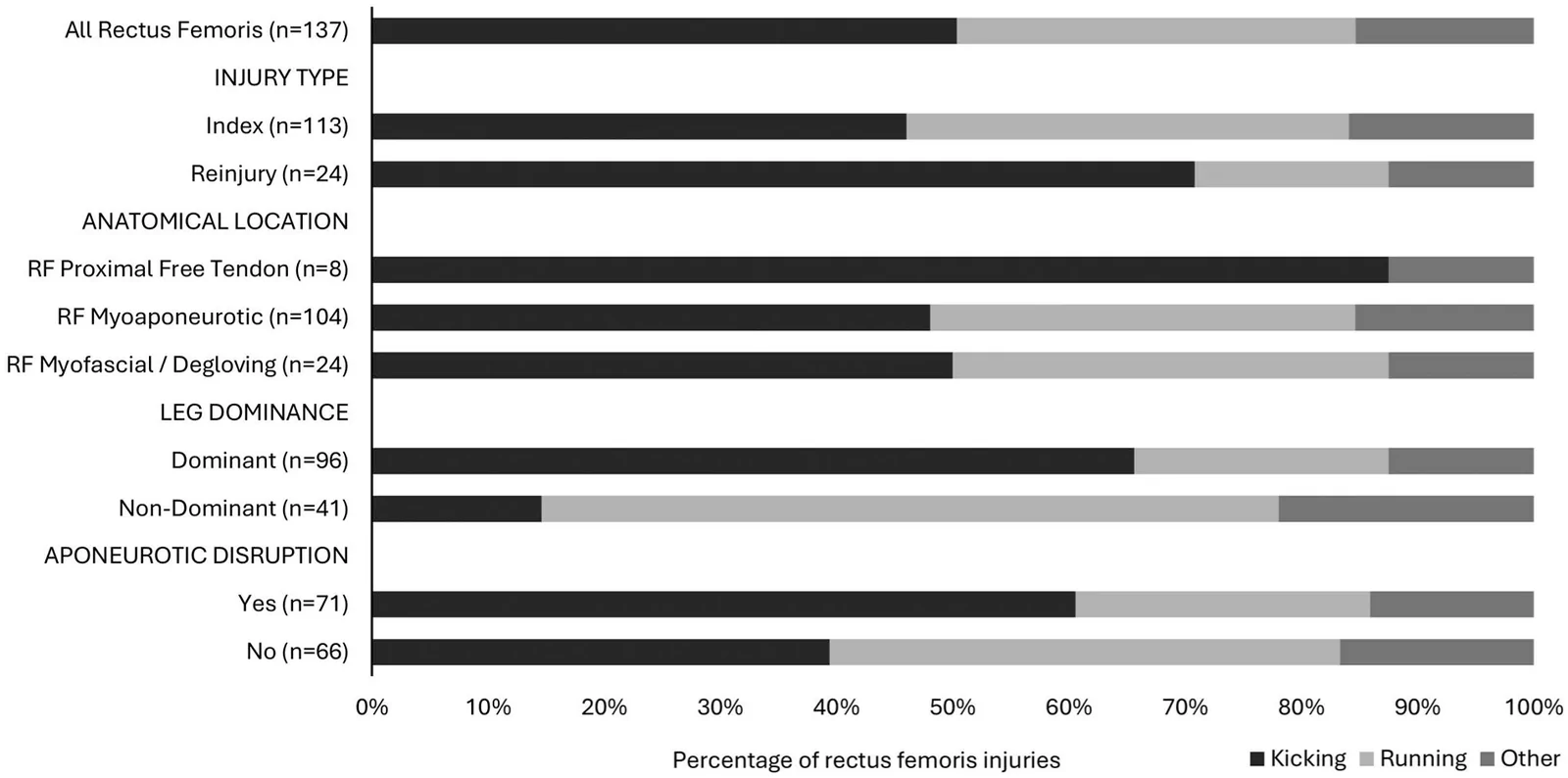
Mechanism of injury with reference to injury type, anatomical location, leg dominance, and presence of AD. AD, aponeurotic disruption.

A total of 69 (46.9%) rectus femoris injuries occurred from a kicking mechanism, and 47 (32%) had a specific running-based mechanism (Figure 1). Nearly all (87.5%) rectus femoris proximal free tendon injuries were from a kicking mechanism. Injuries with AD were more likely to occur from a kicking mechanism when compared with running or other injury mechanisms (*χ*^2^(2) = 6.6; *P* = 0.04). A kicking mechanism was more common for reinjuries compared with index injuries (*χ*^2^(1) = 4.9; *P* = 0.03).

#### Reinjuries

**Figure 2.**
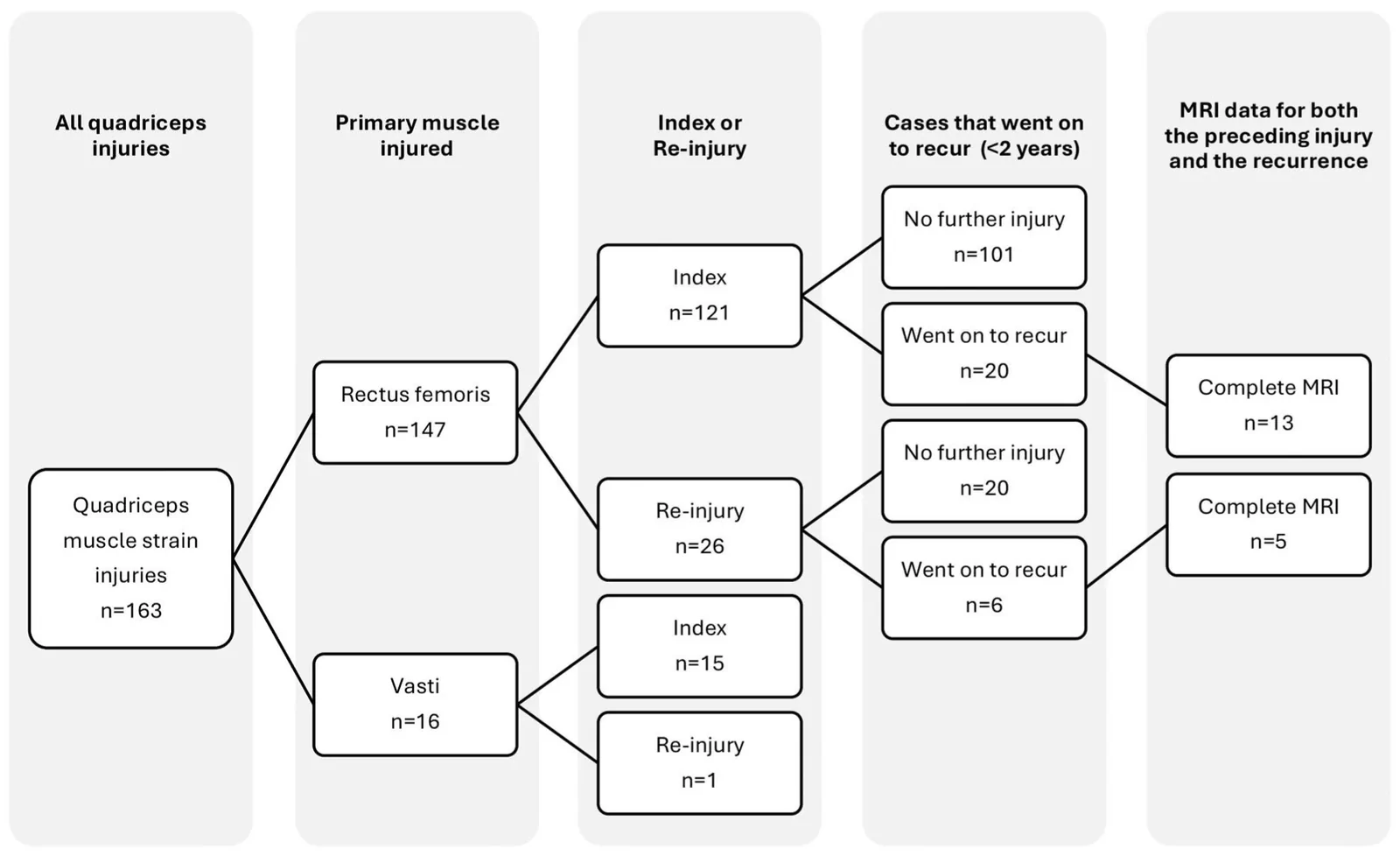
Number of cases by primary muscle injured, injury type, and recurrence. MRI, magnetic resonance imaging.

A total of 26 cases were categorized as rectus femoris reinjuries (Figure 2). The median time to reinjury was 108 days (IQR = 220). Two-thirds of reinjuries (66.6%) occurred in the same season. In all, 11 (40.7%) cases were early reinjuries, occurring within 2 months; 9 (81.8%) early reinjuries were myoaponeurotic, while the other 2 were isolated degloving injuries.

#### Cases That Went on to Recur

**Figure 3.**
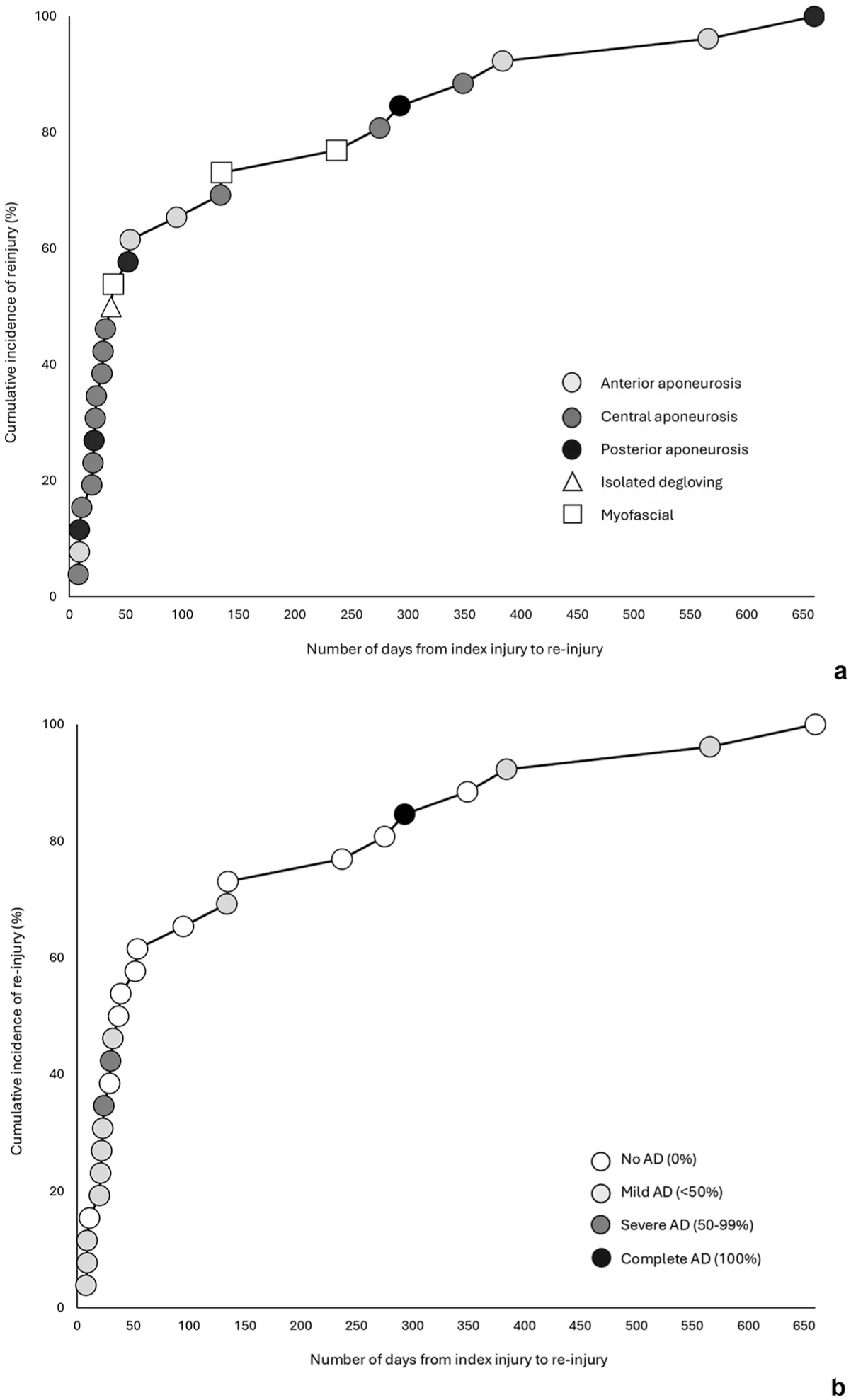
The cumulative incidence of reinjury. (a) Primary anatomical location of the index injury. (b) Presence and severity of AD. AD, aponeurotic disruption.

Throughout the 9-season data collection period, 26 rectus femoris injuries went on to recur within 2 years (Figure 2). These were most commonly myoaponeurotic injuries (n = 21, 80.7%) with nearly half involving the central aponeurosis (n = 12, 46.2%). Cases that went on to recur early (n = 16, 59.3%) were predominantly myoaponeurotic injuries (n = 14, aponeurosis: central n = 9, posterior n = 3, anterior n = 2), while 1 case was a degloving injury (Figure 3).

Complete MRI details for both the preceding injury and the recurrence were available for 18 cases (Figure 2). In 11 (61.1%) cases, the preceding injury and the recurrence affected the same general position and anatomical location, whereas in 3 cases the recurrence affected the same general position but a different anatomical location, and for 5 cases the recurrence involved a different general position and a different anatomical location. Recurrences usually had the same (n = 9, 50%) or worse (n = 6, 33.3%) severity of AD relative to the preceding injury.

#### Degloving Injuries

Degloving was reported in 14 cases. Of these 14 cases, 6 (42.9%) were isolated degloving injuries. Half (50%, n = 7) had a concurrent myoaponeurotic injury (central aponeurosis n = 5, anterior aponeurosis n = 1, posterior aponeurosis n = 1). Seven degloving injuries had retraction of the inner bipennate muscle belly, with a median of 10 mm of retraction (range, 8-25 mm). Three (21.4%) degloving injuries went on to recur at a mean of 30 days postinjury (median, 29 days; range, 24-37 days). Two of these had a concurrent injury to the central aponeurosis.

### Functional Progression and RTP

#### Anatomical Location

**Figure 4.**
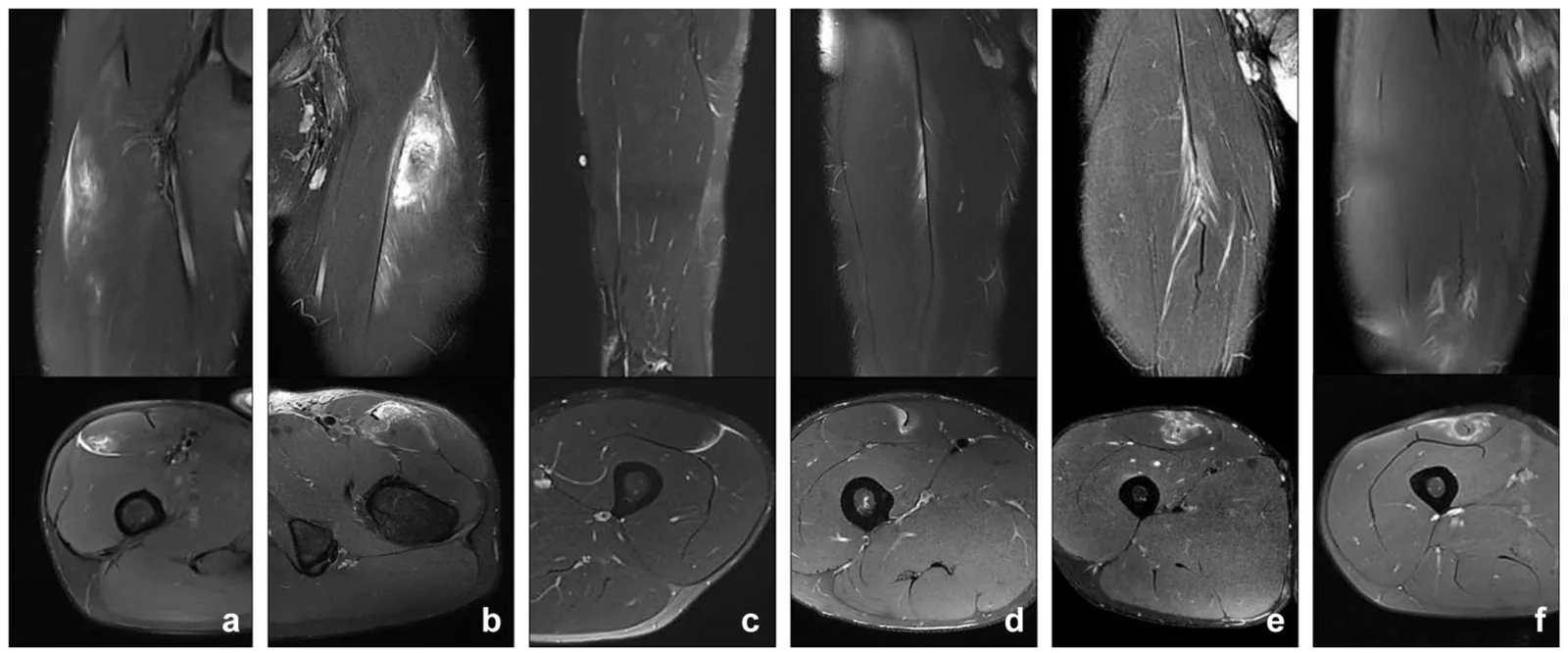
MRI of rectus femoris injuries located at various anatomical locations. (a) Posterolateral myofascial injury with intermuscular hematoma. (b) Anterior aponeurosis strain with mild AD and intramuscular hematoma. (c) Posterior lateral aponeurosis strain with mild AD. (d) Central aponeurosis myoaponeurotic strain with no AD. (e) Central aponeurosis strain with severe AD and waviness. (f) Primary degloving injury with retraction and associated waviness of central aponeurosis. AD, aponeurotic disruption; MRI, magnetic resonance imaging.

The anatomical location of rectus femoris injury (Figure 4) was associated with the time taken to RTP (*χ*^2^(3) = 12.76; *df* = 3; *P* = 0.01). When compared with other locations, free tendon injuries took the longest time to RTP (50.4 days ± 16.2). Central aponeurosis injuries took longer to RTP (33.7 days ± 20.4) compared with injuries involving the anterior aponeurosis (27.7 days ± 13.5; B = –0.53; HR, 0.59; 95% CI, 0.35-0.99; *P* = 0.05) and posterior aponeurosis (26.6 days ± 12.7; B = –0.58; HR, 0.56; 95% CI, 0.35-0.91; *P* = 0.02). Vasti injuries required on average 14.5 days (±8.4) to RTP (Table 2; Supplementary File 2 and Table S3, available online).

**Table 2.** Time (days) to reach functional progression milestones for each anatomical location of injury with and without AD

	Return to pain-free walking			Return to run at >90% of maximum speed			Return to full training			RTP		
	N	Mean ± SD	Median (IQR)	N	Mean ± SD	Median (IQR)	N	Mean ± SD	Median (IQR)	N	Mean ± SD	Median (IQR)
All rectus femoris	130	2.5 ± 2.3	2 (1-3)	122	21.9 ± 14.1	19.5 (12-26.25)	122	26.9 ± 16.3	24 (15-33)	127	30.3 ± 16.7	28 (20-38)
Free tendon	6	4.3 ± 3.1	4 (1.75-6.25)	7	42.7 ± 18.7	35 (27-65)	6	50 ± 25.6	45.5 (28.5-76.5)	7	50.4 ± 16.2	46 (40-61)
All myoaponeurotic	101	2.7 ± 2.4	2 (1-4)	92	21.5 ± 13.7	19 (12-25)	93	26.6 ± 15.8	24 (15-32.5)	96	30.1 ± 17	27.5 (20-36.75)
With AD	62	2.9 ± 2.5	2 (1-4)	55	23.9 ± 14.9	21 (14-30)	57	28 ± 17.4	25 (18.5-34)	57	32.7 ± 19.1	28 (21-38)
No AD	39	2.5 ± 2.2	2 (1-3)	37	18.1 ± 10.9	16 (9.5-22.5)	36	23.6 ± 13.1	19 (14-31)	39	26.5 ± 12.9	21 (17-33)
Anterior aponeurosis	21	2.1 ± 1.7	2 (1-3)	19	24.1 ± 16.4	21 (15-31)	22	25.3 ± 14.6	23.5 (18.75-30.25)	23	27.7 ± 13.5	28 (21-34)
With AD	16	2.1 ± 1.8	2 (1-3)	15	25.2 ± 17.7	21 (15-31)	17	26 ± 15.4	24 (21-30)	18	28.7 ± 14	28 (21-34)
No AD	5	2 ± 1.2	2 (2-3)	4	20 ± 11.5	19 (12.5-27.5)	5	23 ± 12.9	21 (18-27)	5	24 ± 12	21 (21-27)
Central aponeurosis	51	3.2 ± 2.4	3 (1-5)	45	21.8 ± 14.2	19 (10-26)	45	28 ± 18.2	26 (14.5-38)	44	33.7 ± 20.4	28 (20-44.75)
With AD	35	3.5 ± 2.7	3 (1-5)	30	24.2 ± 14.8	21 (14-31)	30	30.2 ± 20	27.5 (18-40)	29	36.9 ± 23	28 (21-52)
No AD	16	2.5 ± 1.6	2 (1-3.5)	15	16.9 ± 11.7	12 (8-24)	15	23.5 ± 12.5	20 (14-32)	15	27.5 ± 12.7	21 (18-40)
Posterior aponeurosis	29	2.2 ± 2.6	2 (0-3)	28	19.4 ± 10.7	17 (13-24.5)	26	24 ± 12.6	24.5 (13.5-30)	29	26.6 ± 12.7	26 (16-34.5)
With AD	11	1.6 ± 1.9	1 (0-3)	10	20.9 ± 11.1	19 (13-27)	10	24.3 ± 9.4	26.5 (21-29)	10	27.2 ± 10.4	28 (25-38)
No AD	18	2.5 ± 2.9	2 (1-3)	18	18.7 ± 10.7	16.5 (13-22)	16	23.8 ± 14.3	18 (13-30.5)	19	26.4 ± 13.8	22 (13-33)
Myofascial	19	1.4 ± 0.8	1 (1-2)	20	16.8 ± 8.3	17.5 (8-24)	19	21.4 ± 10.9	21 (11-31)	19	24.2 ± 10.6	26 (14-32)
Isolated degloving	4	1 ± 0.8	1 (0.25.1.75)	3	19.7 ± 3.8	18 (17-18)	4	27.5 ± 8.9	26.5 (19.5-36.5)	5	30.2 ± 10.1	26 (23-39.5)
Vastii	16	2.5 ± 3	2 (0.25-3)	15	9.1 ± 4.01	9 (6-12)	16	13.6 ± 12.5	10 (5.5-17)	16	14.5 ± 8.4	12.5 (10.25-19.5)

AD; aponeurosis disruption; RTP, return to play.

#### Mechanism of Injury

Rectus femoris injuries that occurred when kicking took longer to RTP (median, 34 days; IQR, 22-44) compared with rectus femoris injuries from all other combined nonkicking mechanisms (running and other: median 27 days; IQR, 18-34; B = –0.37; HR 0.69; 95% CI, 0.48-0.99; *P* = 0.04) (Supplementary File 2 and Table S4, available online).

#### Injury Severity

Survival analysis revealed rectus femoris injuries with AD present (yes/no) took longer to RTP (yes: median, 33 days; IQR, 25-46 vs no: median, 26 days; IQR, 18-33). Injuries with AD retraction (yes: median, 41 days; IQR, 27-79 vs no: median, 28 days; IQR, 20-38; B = –0.95; HR, 0.39; 95% CI, 0.22-0.69; *P* = 0.001) or waviness (yes: median, 41 days; IQR, 22-63 vs no: median, 28 days; IQR, 20-36; B = –0.81; HR, 0.44; 95% CI, 0.28-0.69; *P* < 0.001) took longer to RTP compared with cases with no evidence of these findings (Supplementary File 2 and Table S4, available online). Further analysis into specific anatomical location of injury revealed central aponeurosis injuries with AD took longer to RTP (median, 33 days; IQR, 23-56) compared with central aponeurosis with no AD (median, 21 days; IQR, 18-40) (B = –0.75; HR, 0.47; 95% CI, 0.25-0.92; *P* = 0.03). This same relationship was not found for anterior (B = –0.39; HR, 0.68; 95% CI, 0.25-1.89; *P* = 0.46) or posterior (B = –0.14; HR, 0.87; 95% CI, 0.39-1.9; *P* = 0.73) aponeurosis injuries with and without AD (Supplementary File 2 and Table S3, available online).

The presence of an intramuscular hematoma also showed a univariate association with worse recovery (return to full training: B = –0.42; HR, 0.65; 95% CI, 0.45-0.95; *P* = 0.02; RTP: B = –0.44; HR, 0.65; 95% CI, 0.45-0.93; *P* = 0.02). In cases where degloving occurred, time to RTP was not affected by retraction (yes/no) (*χ*^2^(1) = 0.47; *P* = 0.49), or between isolated degloving injuries and those that had concurrent myoaponeurotic injuries (*χ*^2^(1) = 0.01; *P* = 0.92).

#### Reinjuries

Compared with index injuries, cases categorized as rectus femoris reinjuries took longer to return to full training (B = –0.88; HR, 0.42; 95% CI, 0.25-0.70; *P* = 0.001) and RTP (B = –0.82; HR, 0.44; 95% CI, 0.26-0.74; *P* = 0.002) (Table 3).

**Table 3.** Clinical and MRI data associated with return to full training and RTP after rectus femoris muscle strain

	Return to full training						RTP					
	Univariate			Multivariable			Univariate			Multivariable		
	B	HR (95% CI)	*P* value	B	AHR (95% CI)	*P* value	B	HR (95% CI)	*P* value	B	AHR (95% CI)	*P* value
Age, years	0.01	1.01 (0.96-10.6)	0.73	0.03	1.03 (0.98-1.08)	0.31	0.15	1.02 (0.97-1.06)	0.53	0.05	1.05 (1.0-1.1)	0.06
Reinjury	–0.88	0.42 (0.25-0.7)	0.001*	–0.87	0.42 (0.21-0.82)	0.01*	–0.82	0.44 (0.26-0.74)	0.002*	–0.7	0.49 (0.26-0.96)	0.04*
Previous QMSI (same side)	–0.42	0.66 (0.45-0.97)	0.03*	–0.10	0.90 (0.55-1.47)	0.68	–0.36	0.70 (0.47-1.02)	0.06	–0.07	0.93 (0.58-1.49)	0.76
Kicking MOI	–0.14	0.87 (0.6-1.23)	0.45	0.06	1.06 (0.69-1.64)	0.78	–0.37	0.69 (0.48-0.99)	0.04*	–0.28	0.76 (0.5-1.15)	0.20
Intramuscular hematoma	–0.42	0.65 (0.45-0.95)	0.02*	–0.28	0.76 (0.48-1.18)	0.22	–0.44	0.65 (0.45-0.93)	0.02*	–0.29	0.75 (0.48-1.15)	0.19
Central location	–0.47	0.63 (0.42-0.92)	0.02*	–0.09	0.91 (0.57-1.46)	0.70	–0.54	0.58 (0.4-0.85)	0.006*	–0.16	0.85 (0.55-1.33)	0.48
Severity of AD*^a^*												
Mild AD (<50%)	–0.13	0.88 (0.58-1.32)	0.52	–0.21	0.81 (0.52-1.27)	0.36	–0.23	0.80 (0.53-1.2)	0.28	–0.28	0.76 (0.49-1.17)	0.21
Severe AD (50% to 99%)	–0.73	0.48 (0.27-0.86)	0.01*	–0.55	0.58 (0.28-1.18)	0.13	–0.96	0.38 (0.21-0.66)	<0.001*	–0.76	0.47 (0.24-0.93)	0.03*
Complete AD (100%)	–1.77	0.17 (0.07-0.41)	<0.001*	–1.81	0.16 (0.06-0.43)	<0.001*	–2.01	0.13 (0.06-0.32)	<0.001*	–2.02	0.13 (0.05-0.34)	<0.001*

AD, aponeurotic disruption; AHR, adjusted hazard ratio; HR, hazard ratio; MOI, mechanism of injury; MRI, magnetic resonance imaging; QMSI, quadriceps muscle strain injury; RTP, return to play.

a Severity of AD compared with cases with no AD (Mild AD [<50%] vs No AD, Severe AD [50% to 99%] vs No AD, Complete AD [100%] vs No AD).

* Statistically significant association with outcome (*P* < 0.05).

#### Multivariable Analysis

When clinical and MRI findings were evaluated together for all rectus femoris injuries, multivariable analysis showed increased severity of AD (severe AD: B = –0.76; adjusted HR [AHR], 0.47; 95% CI, 0.24-0.93; *P* = 0.03; complete AD: B = –2.20; AHR, 0.13; 95% CI, 0.05-0.34; *P* < 0.001) and reinjury (B = –0.7; AHR, 0.49; 95% CI, 0.26-0.96; *P* = 0.04) were the only factors associated with longer RTP (Table 3).

#### Injury Recurrence

Univariate analysis did not reveal any associations between clinical or MRI findings of the preceding rectus femoris injury and recurrence, for either early recurrences (within 2 months) or all recurrences (within 2 years) (Supplementary File 2 and Table S5, available online). The time taken to RTP after rectus femoris injury was not associated with risk of recurrence (B = –0.08; HR, 0.92; 95% CI, 0.59-1.45; *P* = 0.73).

## Discussion

This study reports key findings on RTP outcomes after quadriceps muscle strain injuries in elite male Australian football players. First, the mechanism of injury is associated with the location and severity of rectus femoris injury. Second, the presence and severity of connective tissue damage, particularly in the central aponeurosis of rectus femoris, is associated with prolonged recovery. Third, rectus femoris reinjuries are associated with longer RTP. Finally, no clinical or MRI findings were related to the risk of recurrence. These results provide detailed guidance on expected recovery timeframes after quadriceps muscle strain and highlight specific injury subsets that may require extended rehabilitation.

RTP duration was impacted by the anatomical location of injury, with injuries in the proximal free tendons (mean, 50.4 ± 16.2 days) and central aponeurosis (mean, 33.7 ± 20.4) of rectus femoris requiring the longest time to RTP. Our findings are consistent with findings in hamstring strain injuries where tendon involvement (i.e., free tendon or intramuscular tendon and/or aponeurosis) is associated with prolonged time to RTP.^11,43,44,48,49^

Severe rectus femoris injuries affecting the direct and indirect free tendons of rectus femoris may require surgical treatment to preserve function and decrease incidence of reinjuries.^3,27,29,41^ In our cohort there were no surgical cases: 4 complete free tendon ruptures RTP in a mean 59.3 ± 23.5 days (median, 61 days; range, 35-82 days) without surgery or recurrence. This is consistent with findings from a recent systematic review on management of proximal rectus femoris injuries in soccer, American football, and track athletes,^3^ although their conservative treatment group reported longer return to sport timeframes (mean, 14.5 (4-36) weeks). It is possible that included studies represented a biased sample of more severe injuries from case series (n = 13 studies) and case reports (n = 25 studies).

Our analysis identified rectus femoris proximal free tendon injuries occurred from kicking, with no cases occurring from running-related mechanisms. Giess Santos et al^16^ similarly linked kicking to proximal free tendon injuries in a large soccer cohort (n = 105). Their reported injury mechanisms showed similar percentages to our study (kicking 54.3% vs 49.6%; running 31.3% vs 33.8%), although they reported a higher percentage of proximal free tendon injuries (19% vs 5.5%). Proximal free tendon injuries have been reported to occur in elite track athletes, possibly due the higher sprint velocities of the elite track athlete relative to soccer or Australian rules players.^30^

Injuries with a kicking mechanism were also associated with connective tissue disruption (presence of AD), which has ramifications for injury prognosis and rehabilitation. Rectus femoris injuries with AD required longer time to reach all recovery milestones (running at 90% of maximum speed, return to full training, RTP). These results are consistent with outcomes in calf strain injuries,^18,45,47^ although evidence for hamstring strain injuries is mixed.^2,26^ The extended recovery in cases with AD may reflect the increased time required for connective tissue healing and scar remodeling. A delayed return of tensile strength and stiffness (required for high load activities such as sprinting or kicking) in the recently injured tissue may be expected, resulting in extended rehabilitation to restore optimal function.^21,34^

A greater severity of AD was associated with longer recovery, consistent with previous research in other muscle injuries where high-grade AD has been linked to a worse prognosis.^4,11,18,37,44,45^ A recent systematic review (hamstring, n = 378; quadriceps, n = 63) reported the percentage CSA of tendon disruption, waviness, and loss of tendon tension on MRI (all markers of increased severity of connective tissue damage) showed moderate evidence for an association with increased time to RTP.^2^

We have demonstrated that rectus femoris injuries with disruption to the central aponeurosis took longer to RTP than injuries at the myoaponeurotic junction with no AD, and longer than all injuries at the anterior or posterior aponeuroses, regardless of whether AD was present. This is consistent with findings in track athletes where conservatively managed ‘central tendon’ injuries (i.e., central aponeurosis) took longer time to return to full training than myofascial injuries.^30^ Previous studies in soccer and Australian rules football have found that injuries involving the intramuscular component of the central aponeurosis require longer recovery periods,^1,7^ and may require different management compared with peripheral injuries.^4^ The central aponeurosis provides a critical element to allow force transmission through the rectus femoris, acting as a central supporting strut for bipennate muscle fibers along its trajectory, particularly when actively lengthening under eccentric load (for example, in the cocking phase of kicking, or when initiating swing in sprinting).^38^ Disruption of the central aponeurosis may therefore interrupt the integrity of the rectus femoris muscle-tendon network, and impede its capacity to tolerate high loads.^34^

In this study, mean RTP time frames for isolated (30.2 ± 10.1 days) or concurrent (24.4 ± 13.9 days) degloving injuries were not significantly different to myoaponeurotic (30.1 ± 17 days) or myofascial (24.2 ± 10.6 days) rectus femoris injuries; however, our sample size was limited (n = 6 isolated, n = 14 total degloving cases). These results challenge current perception that degloving results in longer recovery.^25,31,32^ Rectus femoris degloving injuries are caused by the unique anatomy of the rectus femoris inner bipennate and outer unipennate fiber distribution, which creates a “muscle within a muscle” phenomenon,^25,38^ and are therefore described poorly by current grading systems. The extent of AD in degloving injuries may be the most salient finding related to prognosis, although further analysis in a larger sample is required.

It should be acknowledged that other factors, such as clinical presentation, severity of symptoms, demands of the sport, playing position, stage of the season, and player psychology, should also be considered when estimating recovery after injury.^9^ The large variation in RTP days for injuries with the same anatomical location and severity in this study (e.g., central aponeurosis injuries with AD, 9 to 98 days), highlights the need to consider clinical, imaging, and contextual information in the RTP decision-making process.

No associations between clinical or MRI findings at the preceding injury and risk of recurrence were found for either early recurrences (within 2 months) or all recurrences (within 2 years). High-grade rectus femoris injuries may be more prone to recurrence,^30^ while myofascial injuries seem less likely to reinjure than other injury classes,^24,38^ although several studies have identified that baseline MRI findings were not useful for predicting the risk of recurrence.^8,22,50^ Of clinical interest, all cases that went on to recur within the first month (30 days, n = 13) were rectus femoris myoaponeurotic injuries, with 12 of 13 showing AD. Pathoanatomical differences between muscle and aponeurosis tissue healing may need to be considered when developing rehabilitation and ongoing prevention programs for rectus femoris injuries with and without AD.

This is the largest study to evaluate the associations between clinical and MRI findings with RTP and recurrence after quadriceps muscle strain injuries in an elite cohort. Study strengths include a single sport cohort, with consistent match and training schedules, and unique playing and training demands. Radiologists were blinded to clinical data when evaluating deidentified MRI scans. While this study provides valuable insights, some study limitations should also be acknowledged. Clinical data were submitted by different staff from participating teams. Rehabilitation was completed by treating medical teams that were not blinded to MRI findings, which likely influenced rehabilitation decisions. Nevertheless, this study involved elite-level athletes and high-performance teams all aiming for RTP as soon as possible. Data were collected only for injured players, with exposure data not available. In addition, the sample sizes in some injury categories, combined with the collection of data from a single sport with only male athletes may limit the generalizability of the results. Further research is needed to validate these findings in a larger cohort, with a more diverse sporting population, including female athletes.

## Conclusion

Rectus femoris injuries with a greater severity of connective tissue damage are associated with a longer RTP. Longer rehabilitation timeframes may be required for rectus femoris injuries involving the proximal free tendons and central aponeurosis. Cases with disruption of the central aponeurosis may require longer time to RTP than central aponeurosis injuries with no disruption, and longer than all injuries at the anterior or posterior aponeuroses. Reinjuries took longer to RTP, but we did not find any baseline clinical or imaging findings to be associated with an increased risk of recurrence.

### Clinical Recommendations

- Recovery timeframes after rectus femoris injury are thought to be influenced by the anatomical location and extent of connective tissue disruption. However, there is limited evidence exploring the association between clinical and imaging findings after rectus femoris strain with prognosis and reinjury.
- The anatomical location and severity of rectus femoris injury was associated with the time taken to RTP. Injuries involving the proximal free tendon or the central aponeurosis of rectus femoris, injuries with more significant connective tissue disruption, and reinjuries of the rectus femoris required extended time to RTP. No clinical or MRI factors concerning the initial injury were associated with injury recurrence.
- An understanding of baseline MRI findings related to the specific anatomical location and severity of connective tissue disruption can enhance the ability of a clinician to estimate RTP timeframes after rectus femoris injury.
- Strength of Recommendation Taxonomy (SORT): B (https://www.aafp.org/assets/image/upload/v1746724644/sort_oz8ecz.pdf).

## Supplemental Material

sj-docx-1-sph-10.1177_19417381261476889 – Supplemental material for Imaging and Clinical Factors Associated With Return to Play and Recurrence After Quadriceps Muscle Strain Injury Supplemental material, sj-docx-1-sph-10.1177_19417381261476889 for Imaging and Clinical Factors Associated With Return to Play and Recurrence After Quadriceps Muscle Strain Injury by Samuel Pietsch, Brady Green, Anthony G. Schache, Andrew Rotstein, Milo MacBain, Michael Paks and Tania Pizzari in Sports Health

sj-docx-2-sph-10.1177_19417381261476889 – Supplemental material for Imaging and Clinical Factors Associated With Return to Play and Recurrence After Quadriceps Muscle Strain Injury Supplemental material, sj-docx-2-sph-10.1177_19417381261476889 for Imaging and Clinical Factors Associated With Return to Play and Recurrence After Quadriceps Muscle Strain Injury by Samuel Pietsch, Brady Green, Anthony G. Schache, Andrew Rotstein, Milo MacBain, Michael Paks and Tania Pizzari in Sports Health
