# Supplementary material for: Imaging and Clinical Factors Associated With Return to Play and Recurrence After Quadriceps Muscle Strain Injury
Source: Sports Health. 2026 Aug 27:19417381261476889. Online ahead of print. doi: 10.1177/19417381261476889 (PMC13525106; doi:10.1177/19417381261476889)
Supplement: Supplementary file 1 [file sj-docx-1-sph-10.1177_19417381261476889.docx]

Quadriceps Injury MRI Study

PLAYER IMAGING ID NUMBER:	__________________________________
Reviewing radiologist	◯ Andrew Rotstein<br>◯ Milo Macbain<br>◯ Michael Paks

QUADRICEPS MUSCLE INJURED

Primary muscle injured?<br><br>**If more than one muscle is injured, the muscle with the largest injury and/or severity of disruption should be classified as the primary muscle involved.	◯ Rectus Femoris<br>◯ Vastus medialis<br>◯ Vastus intermedialis<br>◯ Vastus Lateralis<br>◯ Other (please describe)
Please describe other location	__________________________________
Was there a second quadriceps muscle injured?	◯ Yes<br>◯ No

LOCATION OF PRIMARY INJURY

Location - proximal to distal<br>(classify the location of the injury within the primary muscle, split rectus femoris muscle into approximate proximal, middle and distal thirds from origin to insertion)	◯ Proximal third<br>◯ Middle third<br>◯ Distal third
Please record the distance of the injury from the AIIS (in millimetres)<br><br>(If not possible please record as N/A)	__________________________________
Please record the distance of the injury from the superior margin of the patella (in millimetres)<br><br>(If not possible please record as N/A)	__________________________________
Location - depth<br>(classify the depth of the injury within the primary muscle, split rectus femoris muscle into approximate anterior (superficial) and posterior (deep) halves)	◯ Superficial<br>◯ Deep<br>◯ Both superficial and deep
Location - medial to lateral<br>(classify the location of the injury within the primary muscle, split rectus femoris muscle into approximate medial, central and lateral thirds from medial to lateral borders of the muscle)	◯ Medial<br>◯ Central<br>◯ Lateral

Anatomical Location of Injury? (RECTUS FEMORIS)<br><br>**If there is more than one anatomical location of injury, multiple loctions of injury can be selected<br><br>**the site with the largest injury and/or severity of disruption will be classified as the primary anatomical location	☐ Free Tendon - Proximal direct tendon - Proximal indirect tendon - Proximal conjoint tendon - Distal quadriceps tendon<br>☐ Musculotendinous Injury * Injury to connection between muscle fibres and an aponeurosis or a tendinous expansion - Anterior aponeurosis - Central aponeurosis / central tendon - Posterior aponeurosis<br>☐ Myofascial Injury * Injury to connection between muscle fibres and the epimysium / perimysium or an epimyseal / fascial injury)<br>☐ Intramuscular Injury * Muscle fibre injury (DOMS, contusion)<br>☐ Degloving injury *Partial or complete dissociation between the inner muscle and outer muscle belly with or without retraction
Anatomical Location of Injury (VASTII)<br><br>**If there is more than one anatomical location of injury, the site with the largest injury and/or severity of disruption should be classified as the primary anatomical location	☐ Distal free tendon - quadriceps tendon<br>☐ Musculotendinous Junction Injury * injury to connection between muscle fibres and an aponeurosis or a tendinous expansion<br>☐ Myofascial Injury * connection between muscle fibers and the epimysium / perimysium or an epimysial / fascial injury

Description of Injury Severity

Location of free tendon injury?	◯ Proximal direct tendon<br>◯ Proximal indirect tendon<br>◯ Proximal conjoined tendon<br>◯ Distal quadriceps tendon
Is there evidence of free tendon disruption (Free tendon disruption defined as fluid signal intensity partially or completely disrupting the tendon on T2 weighted images)	◯ Yes<br>◯ No
Severity of free tendon disruption (% scale)	◯ None (0%)<br>◯ Mild (< 50%)<br>◯ Severe (between 50%-99%)<br>◯ Complete (100% of the structural cross-sectional area)
Please describe the longitudinal length of tendon injury (length of tendon which has signal abnormality in millimetres).	________________________________
Presence of waviness / loss of tendon tension?	◯ Yes<br>◯ No
Free tendon retraction?	◯ Yes<br>◯ No
Please record the measurable gap / defect in the tendon (in millimetres).	________________________________

Location of musculotendinous injury.	◯ Anterior aponeurosis * extension of direct head, proximal third of RF<br>◯ Central aponeurosis / central tendon * extension of indirect head, proximal and middle third of RF<br>◯ Posterior aponeurosis * extension of distal tendon, middle and distal third of RF<br>◯ Other
Please describe location of other musculotendinous injury.	__________________________________
Is there evidence of aponeurosis disruption?<br><br>(Aponeurosis disruption defined as fluid signal intensity partially or completely disrupting the aponeurosis on T2 weighted images, an irregular wavy contour or focal defect separating proximal and distal parts of the aponeurosis)	◯ Yes<br>◯ No
Severity of aponeurosis disruption (% scale)	◯ None (0%)<br>◯ Mild (< 50%)<br>◯ Severe (between 50%-99%)<br>◯ Complete (100% of the structural cross-sectional area)
Please describe the longitudinal length of tendon injury (length of tendon which has signal abnormality in millimetres).	__________________________________
Presence of waviness / loss of tension?	◯ Yes<br>◯ No
Aponeurosis retraction?	◯ Yes<br>◯ No
Please record the measurable gap / defect in the aponeurosis (in millimetres).	__________________________________
Myofascial injury location.	◯ Anterior epimysium<br>◯ Posterior epimysium<br>◯ Other
Description of intramuscular injury.	◯ DOMS<br>◯ Contusion<br>◯ Other
Please describe the intramuscular injury.	__________________________________

DEGLOVING INJURY

Is there a degloving injury to rectus femoris?<br><br>** Degloving injury defined as a partial or complete dissociation between the inner muscle belly from the outer muscle with or without associated retraction	◯ Yes<br>◯ No
Is there retraction of the inner muscle belly?	◯ Yes<br>◯ No
If possible, please record the measure of retraction (in millimetres)<br><br>(If unable please record as N/A)	________________________

IMAGING FEATURES

Presence of muscule oedema?	◯ Yes<br>◯ No
Oedema length (maximal craniocaudal length in millimeters)	________________________
Oedema width (maximal transverse width in millimetres)	________________________
If possible please record the length of fibre disruption (maximum craniocaudal length in mm)<br><br>(if unable please record as N/A)	________________________
Is there INTRA muscular haematoma present?	◯ Yes<br>◯ No
Is there INTER muscular haematoma present?	◯ Yes<br>◯ No
Please describe location	________________________
Is there scar tissue present (ie, from a previous injury)?	◯ Yes<br>◯ No
Where is the scar tissue located?	◯ At the site of acute injury<br>◯ Remote to acute injury

LOCATION OF SECONDARY INJURY

Which second quadriceps muscle was also injured?	◯ Rectus Femoris<br>◯ Vastus medialis<br>◯ Vastus intermedialis<br>◯ Vastus Lateralis
Location - proximal to distal (secondary injury)<br>(classify the location of the injury within the secondary muscle, split muscle into approximate proximal, middle and distal thirds from origin to insertion)	◯ Proximal third<br>◯ Middle third<br>◯ Distal third

SUMMARY OF QUADRICEPS INJURY.

Please provide a brief descriptive summary of the MRI findings.<br><br>(as would be found in the conclusion of an MRI report)	________________________________

CURRENT GRADING SYSTEMS:

Please classify the quadriceps injury using the British Athletics Muscle Injury Classification (BAMIC) system:

British Athletics Muscle Injury Classification System (BAMIC)	○ 0b: DOMS following unaccustomed exercise. MRI: pathcy high signal change throughout one or more muscles.<br>○ 1a: small myofascial tear. MRI: high signal change at the fascial border with < 10% extension into muscle belly. Craniocaudal length of < 5cm.<br>○ 1b: small MTJ tear. MRI: high signal change that is < 10% cross-section area of muscle at the MTJ. Longitudinal length < 5 cm with < 1 cm fibre disruption.<br>○ 2a: moderate myofascial tear. MRI: high signal at fascial border with extension into muscle. Cross-sectional area between 10-50%; longitudinal length 5-15 cm with < 5 cm fibre disruption.<br>○ 2b: moderate MTJ tear. MRI: high signal at MTJ. Cross-sectional area between 10-50%; longitudinal length 5-15 cm with < 5 cm fibre disruption.<br>○ 2c: moderate sized intratendinous tear. MRI: high signal change extends into the tendon with longitudinal length of tendon involvement < 5cm. Cross-sectional area of tendon involvement < 50% of maximal cross-sectional area.<br>○ 3a: extensive myofascial tear. MRI: high signal change at fascial border that extends into the muscle. >50% cross-sectional area; high signal change of a longitudinal length >15 cm with >5 cm fibre disruption.<br>○ 3b: extensive MTJ tear. MRI: high signal change cross-sectional area of >50%; high signal change of a longitudinal length >15 cm with >5 cm fibre disruption.<br>○ 3c: extensive intratendinous tear. MRI: high signal change extends into tendon. Longitudinal length of tendon invovlement >5cm; Cross sectional area of tendon involvement >50%. There may be loss of tendon tension although no discontinuity is evident.<br>○ 4: full thickness tear of muscle. MRI: complete discontinuity of the muscle with retraction.<br>○ 4c: full thickness tear of tendon. MRI: complete discontinuity of the tendon with retraction.
SCAN LIKELY TO NEED REVIEW FOR CONSENSUS	○ Yes<br>○ No
Please comment if any:<br>- issues with images<br>- limitations with ability to report accurately<br>- reasons for review of images for consensus	________________________________
