# Supplementary material for: Imaging and Clinical Factors Associated With Return to Play and Recurrence After Quadriceps Muscle Strain Injury
Source: Sports Health. 2026 Aug 27:19417381261476889. Online ahead of print. doi: 10.1177/19417381261476889 (PMC13525106; doi:10.1177/19417381261476889)
Supplement: Supplementary file 2 [file sj-docx-2-sph-10.1177_19417381261476889.docx]

**SUPPLEMENTARY FILE 2.**

Supplementary Table S1. Quadriceps muscle involvement for 163 cases

|  | Primary Muscle Injured (N=163) | Secondary Muscle Injured (N=41, 25.2%) |
| --- | --- | --- |
| Rectus femoris | 147 (90.2%) | 30 (18.4%) |
| Free tendon | 9 (5.5%) | 0 (0%) |
| Myo-aponeurotic | 111 (68.1%) | 27 (16.6%) |
| Myofascial | 21 (12.9%) | 3 (1.8%) |
| Degloving | 6 (3.7%) | 0 (0%) |
| Vasti | 16 (9.8%) | 11 (6.8%) |
| Vastus intermedialis | 12 (7.4%) | 9 (5.5%) |
| Vastus medialis | 3 (1.8%) | 2 (1.2%) |
| Vastus lateralis | 1 (0.6%) | 0 (0%) |

Supplementary Table S2. Anatomical location and degree of rectus femoris aponeurotic disruption

|  | None (0%) | Mild (<50%) | Severe (50-99%) | Complete (100%) | Total |
| --- | --- | --- | --- | --- | --- |
| Free Tendon | 0 | 0 | 4 | 5 | 9 |
| Myo-aponeurotic | 42 | 47 | 18 | 4 | 111 |
| Anterior Aponeurosis | 5 | 15 | 4 | 0 | 24 |
| Central Aponeurosis | 16 | 21 | 14 | 4 | 55 |
| Posterior Aponeurosis | 21 | 11 | 0 | 0 | 32 |
| Myofascial | 21 | 0 | 0 | 0 | 21 |
| Isolated degloving | 6 | 0 | 0 | 0 | 6 |

Supplementary Table S3. Estimated mean and median survival days to return to play comparing anatomical location of injury with and without aponeurotic disruption from Kaplan Meier survival analysis

|  | N= | Censored Cases (%) | Mean ± SD | 95% CI of Mean | Median | IQR |
| --- | --- | --- | --- | --- | --- | --- |
| All rectus femoris | 145 | 18 (12.4) | 32.9 ± 1.7 | 29.6-36.1 | 28 | 21-41 |
| Free tendon | 9 | 2 (22.2) | 58.3 ± 7.1 | 44.5-72.1 | 48 | 41-82 |
| All myo-aponeurotic | 110 | 14 (12.7) | 32.0 ± 1.8 | 28.4-35.5 | 28 | 21-40 |
| With AD | 69 | 12 (17.4) | 35.4 ± 2.7 | 30.2-40.6 | 29 | 22-40 |
| No AD | 41 | 2 (4.9) | 26.9 ± 2.1 | 22.8-31 | 22 | 18-35 |
| Anterior aponeurosis | 24 | 1 (4.2) | 27.8 ± 2.8 | 22.3-33.2 | 28 | 21-34 |
| With AD | 19 | 18 (5.3) | 28.8 ± 3.3 | 22.4-35.2 | 28 | 21-34 |
| No AD | 5 | 0 (0) | 24.0 ± 5.4 | 13.5-34.5 | 21 | 21-27 |
| Central aponeurosis | 55 | 11 (20) | 37.3 ± 3.2 | 31.0-43.7 | 30 | 21-52 |
| With AD | 39 | 10 (25.6) | 41.8 ± 4.4 | 33.2-50.5 | 33 | 23-56 |
| No AD | 16 | 1 (6.3) | 28.4 ± 3.3 | 21.9-34.9 | 21 | 18-40 |
| Posterior aponeurosis | 31 | 2 (6.5) | 27.2 ± 2.3 | 22.6-31.7 | 27 | 17-35 |
| With AD | 11 | 1 (9.1) | 28.7 ± 3.1 | 22.5-34.8 | 29 | 25-38 |
| No AD | 20 | 1 (5) | 26.4 ± 3.2 | 20.1-32.6 | 22 | 13-33 |
| Myofascial | 20 | 1 (5) | 25.1 ± 2.5 | 20.2-30 | 26 | 14-32 |
| Isolated degloving | 6 | 1 (16.7) | 30.2 ± 4.5 | 21.4-39 | 26 | 23-32 |
| Vastii | 16 | 0 (0) | 14.5 ± 2.1 | 10.4-18.6 | 12 | 10-15 |

Abbreviations: AD, aponeurosis disruption

Supplementary Table S4. Univariate analysis of clinical and MRI data associated with return to full training, and return to play after rectus femoris muscle strain

|  | Days return to full train | | | Days return to play | | |
| --- | --- | --- | --- | --- | --- | --- |
|  | B | HR (95% CI) | P | B | HR (95% CI) | P |
| Re-injury | -0.88 | 0.42 (0.25-0.7) | 0.001* | 0.82 | 2.26 (0.26-0.74) | 0.002* |
| Age | 0.01 | 1.01 (0.96-1.06) | 0.731 | 0.02 | 1.02 (0.97-1.06) | 0.528 |
| Previous quadriceps strain (any) | -0.33 | 0.72 (0.5-1.03) | 0.073 | -0.30 | 0.74 (0.52-10.5) | 0.095 |
| Previous quadriceps strain (same side) | -0.42 | 0.66 (0.44-0.97) | 0.034* | -0.36 | 0.70 (0.47-1.02) | 0.064 |
| Previous hamstring strain | -0.02 | 0.98 (0.68-1.42) | 0.933 | 0.06 | 1.06 (0.74-1.53) | 0.737 |
| Previous hip Injury | 0.11 | 1.12 (0.69-1.812) | 0.655 | 0.39 | 1.48 (0.93-2.35) | 0.099 |
| Previous knee injury | -0.32 | 0.72 (0.49-1.06) | 0.095 | -0.21 | 0.81 (0.56-1018) | 0.279 |
| Dominant Leg | 0.08 | 1.08 (0.73-1.61) | 0.688 | -0.02 | 0.98 (0.67-1.44) | 0.936 |
| Kicking Mechanism | -0.14 | 0.87 (0.6-1.25) | 0.453 | -0.37 | 0.69 (0.48-0.99) | 0.044* |
| Ethnicity | -0.16 | 0.85 (0.51-1.43) | 0.543 | -0.18 | 1.20 (0.71-2.03) | 0.501 |
|  |  |  |  |  |  |  |
| Secondary Muscle Injury | 0.42 | 1.53 (0.99-2.35) | 0.056 | 0.51 | 1.66 (1.08-2.54) | 0.020* |
| Presence of AD (yes/no) | -0.52 | 0.60 (0.41-0.86) | 0.006* | -0.68 | 0.51 (0.36-0.74) | <0.001* |
| Aponeurosis Retraction (yes/no) | -1.27 | 0.28 (0.15-0.52) | <0.001* | -0.94 | 0.39 (0.22-0.7) | 0.001* |
| Aponeurosis Waviness (yes/no) | -0.76 | 0.47 (0.3-0.73) | 0.001* | -0.81 | 0.44 (0.29-0.69) | <0.001* |
| Oedema Length (craniocaudal mm) | 0.00 | 1.00 (0.99-1.0) | 0.290 | 0.00 | 1.00 (0.99-1.0) | 0.214 |
| Oedema width (transverse mm) | -0.01 | 0.99 (0.97-1.0) | 0.133 | -0.01 | 0.99 (0.98-1.01) | 0.203 |
| Intramuscular Haematoma | -0.42 | 0.65 (0.45-0.95) | 0.024* | -0.44 | 0.65 (0.45-0.93) | 0.019* |
| Intermuscular Haematoma | -0.32 | 0.72 (0.5-1.04) | 0.081 | -0.28 | 0.76 (0.53-1.08) | 0.128 |
| Scar tissue from previous injury | -0.03 | 0.97 (0.68-1.39) | 0.887 | -0.08 | 0.93 (0.65-1.32) | 0.671 |
| Central Location | -0.47 | 0.63 (0.42-0.92) | 0.018* | -0.54 | 0.58 (0.4-0.85) | 0.006* |
| Severity of AD |  |  |  |  |  |  |
| Mild AD (<50%) | -0.13 | 0.88 (0.58-1.32) | 0.523 | -0.22 | 0.8 (0.54-1.23) | 0.275 |
| Severe AD (50-99%) | -0.73 | 0.48 (0.27-0.86) | 0.013* | -0.97 | 0.38 (0.22-0.67) | 0.001* |
| Complete AD (100%) | -1.77 | 0.17 90.07-0.41) | <0.000* | -2.01 | 0.13 (0.06-0.32) | <0.001* |

Supplementary Table S5. Univariate analysis of clinical and MRI data at baseline injury and association with rectus femoris muscle strain recurrence

|  |  | Early (n = 16) | |  | Overall (n = 26) | |
| --- | --- | --- | --- | --- | --- | --- |
|  |  | Univariate | |  | Univariate | |
|  | B | HR (95% CI) | P value | B | HR (95% CI) | P value |
| Age | 0.002 | 1.00 (0.88-1.13) | 0.972 | 0.01 | 1.01 (0.91-1.12) | 0.863 |
| Previous quadriceps strain (any history) | 0.44 | 1.55 (0.56-4.27) | 0.394 | -0.17 | 0.85 (0.35-2.04) | 0.711 |
| Previous quadriceps strain (same side) | 0.31 | 1.36 (0.51-3.65) | 0.544 | -0.64 | 0.53 (0.18-1.57) | 0.251 |
| Previous hamstring Injury | -0.2 | 0.82 (0.31-2.2) | 0.695 | 0-.06 | 0.94 (0.39-2.27) | 0.889 |
| Previous hip Injury | 0.44 | 1.55 (0.49-4.94) | 0.460 | -0.34 | 0.71 (0.21-2.43) | 0.585 |
| Previous knee injury | -0.79 | 0.45 (0.14-1.45) | 0.183 | -0.03 | 0.97 (0.34-2.39) | 0.946 |
| Dominant kicking leg | -0.12 | 0.89 (0.31-2.55) | 0.825 | -0.19 | 0.83 (0.35-1.95) | 0.662 |
| Kicking mechanism | -0.33 | 0.72 (0.25-2.08) | 0.546 | -0.81 | 0.44 (0.17-1.16) | 0.096 |
| Indigenous Australian | -0.06 | 0.94 (0.21-4.15) | 0.937 | 0.32 | 1.37 (0.32-5.86) | 0.668 |
|  |  |  |  |  |  |  |
| Presence of AD (yes/no) | 0.48 | 1.61 (0.58-4.43) | 0.358 | -0.35 | 0.71 (0.31-1.62) | 0.412 |
| Aponeurosis waviness (yes/ no) | -0.36 | 0.70 (0.2-2.46) | 0.579 | 0.02 | 1.02 (0.4-2.6) | 0.959 |
| Oedema length (craniocaudal mm) | 0.001 | 1.00 (0.99-1.01) | 0.803 | 0.00 | 1.00 (1.00-1.01) | 0.210 |
| Intramuscular haematoma | -0.13 | 0.88 (0.32-2.42) | 0.803 | -0.41 | 0.67 (0.27-1.62) | 0.369 |
| Intermuscular haematoma | 0.34 | 1.40 (0.52-3.76) | 0.503 | 0.24 | 1.27 (0.56-2.88) | 0.568 |
| Scar tissue from previous injury | -0.34 | 0.71 (0.26-1.97) | 0.515 | 0.34 | 1.40 (0.62-3.17) | 0.421 |
| Central location | -0.01 | 0.99 (0.34-2.84) | 0.980 | 0.62 | 1.86 (0.63-5.46) | 0.261 |
| Severity of AD |  |  |  |  |  |  |
| Mild AD (<50%) | 0.81 | 2.24 (0.78-6.46) | 0.135 | -0.67 | 0.51 (0.17-1.56) | 0.239 |
| Severe AD (50-99%) | 0.07 | 1.08 (0.22-5.33) | 0.929 | 0.4 | 0.67 (0.19-2.35) | 0.531 |
| Complete AD (100%) | -12.87 | <0.00 (<0.00) | 0.985 | 0.51 | 1.67 (0.48-5.87) | 0.422 |

Abbreviations: AD, aponeurosis disruption, HR, hazard ratio QMSI, quadriceps muscle strain injury.

Proportion of injury type (%)

| Mechanism of Injury | Total by Mechanism Grouped |
| --- | --- |
|  | 49.6403% |
| Kicking (kicking leg) | 49.6403% |
|  | 49.6403% |
| Acceleration | 33.8129% |
| High intensity running | 33.8129% |
| Sudden change of direction | 33.8129% |
| Deceleration | 33.8129% |
| Steady state running | 33.8129% |
| Gradual onset | 16.5468% |
| Nil specific | 16.5468% |
| Other | 16.5468% |
| Stance leg when kicking | 16.5468% |
| Jumping | 16.5468% |

|  | No AD | Mild AD (0-50%) | Severe AD (50-99%) | Complete AD (100%) |
| --- | --- | --- | --- | --- |
|  | 0% | 0% | 0% | 0% |
| Kicking (kicking leg) | 18.705% | 18.705% | 9.35252% | 2.8777% |
|  | 0% | 0% | 0% | 0% |
| Acceleration | 10.0719% | 2.15827% | 1.43885% | 0.719424% |
| High intensity running | 5.76575% | 1.43885% | 2.15827% | 0% |
| Sudden change of direction | 0% | 1.43885% | 0.719424% | 0.719424% |
| Deceleration | 1.43885% | 1.43885% | 0% | 0.719424% |
| Steady state running | 3.59712% | 0% | 0% | 0% |
| Gradual onset | 5.76575% | 1.43885% | 0% | 0% |
| Nil specific | 0.719424% | 0.719424% | 0% | 0% |
| Other | 1.43885% | 0.719424% | 0% | 1.43885% |
| Stance leg when kicking | 1.43885% | 0.719424% | 0% | 1.43885% |
| Jumping | 0% | 0.719424% | 0% | 0% |

Supplementary Figure S1. Mechanism of injury with reference to presence and severity of aponeurotic disruption (AD)

Number of rectus femoris injuries (n)

| Severity of aponeurotic disruption | Waviness | No Waviness |
| --- | --- | --- |
| No AD (0%) | 0 | 69 |
| Mild AD (<50%) | 10 | 37 |
| Severe AD (50-99%) | 18 | 4 |
| Complete AD (100%) | 8 | 1 |

Supplementary Figure S2. The prevalence of waviness with reference to the severity of rectus femoris aponeurotic disruption.

AD; aponeurotic disruption

Supplementary Figure S3. Days to return to play following rectus femoris strain with reference to severity of aponeurosis disruption.
